# Case Report: Large Granular Lymphocyte Leukemia (LGLL)—A Case Series of Challenging Presentations

**DOI:** 10.3389/fonc.2021.775313

**Published:** 2022-01-05

**Authors:** Natali Pflug, Annika Littauer, David Beverungen, Aleksandra Sretenovic, Linus Wahnschaffe, Till Braun, Annika Dechow, Dennis Jungherz, Moritz Otte, Astrid Monecke, Enrica Bach, Georg-Nikolaus Franke, Sebastian Schwind, Madlen Jentzsch, Uwe Platzbecker, Marco Herling, Vladan Vucinic

**Affiliations:** ^1^ Department I of Internal Medicine and Center for Integrated Oncology Aachen Bonn Köln Düsseldorf, University Hospital Cologne, University of Cologne, Cologne, Germany; ^2^ Department of Internal Medicine, GK Mittelrhein, Koblenz, Germany; ^3^ Clinic of Hematology, Cellular Therapy, and Hemostaseology, University of Leipzig, Leipzig, Germany; ^4^ Institute of Hematology, Clinical Center of Serbia, Belgrade, Serbia; ^5^ Institute of Pathology, University of Leipzig, Leipzig, Germany

**Keywords:** LGL leukemia, STAT-3, immunosuppression, NK, TCR, CLPD-NK

## Abstract

Large granular lymphocyte leukemia (LGLL) represents a rare group of diseases with considerable difficulties in their correct diagnostic workup and therapy. The major challenges lie in their distinction from reactive (including autoimmune) lymphoproliferations. Moreover, monoclonal LGL proliferative diseases are in fact a heterogeneous group of disorders, as recognized by the three subtypes in the current WHO classification. It distinguishes two chronic forms (the focus of this case series), namely T-LGLL and chronic lymphoproliferative disorders of Natural Killer cells (CLPD-NK) as well as aggressive NK-cell leukemia. In the clinical routine, the variable presentations and phenotypes of T-LGLL and CLPD-NK are underappreciated. The relevant differential diagnoses range from benign reactive T-cell expansions to other mature T-cell leukemias to highly aggressive γδ-lymphomas. T-LGLL or CLPD-NK patients suffer from a wide variety of symptoms often including, but not limited to, cytopenias or classical autoimmune phenomena. They receive treatments ranging from mere supportive measures (e.g. antibiotics, growth factors, transfusions) over strategies of immunosuppression up to anti-leukemic therapies. The diagnostic pitfalls range from recognition of the subtle T-cell proliferation, repeated establishment of monoclonality, assignment to a descript immunophenotypic pattern, and interpretations of molecular aberrancies. Here, we report a series of selected cases to represent the spectrum of LGLL. The purpose is to raise awareness among the scientifically or practically interested readers of the wide variety of clinical, immunological, and phenotypic features of the various forms of LGLL, e.g. of T-cell type, including its γδ forms or those of NK-lineage. We highlight the characteristics and courses of four unique cases from two academic centers, including those from a prospective nationwide LGLL registry. Each case of this instructive catalogue serves to transport a key message from the areas of (chronic inflammatory) contexts in which LGLL can arise as well as from the fields of differential diagnostics and of various treatment options. Implications for optimization in these areas are discussed.

## Introduction

T-cell large granular lymphocyte leukemia (T-LGLL) is a rare neoplasm, accounting for approximately 2-5% of chronic lymphoproliferative diseases in Western countries. It is characterized by clonal expansion of cytotoxic, often auto-immune reactive, mature T-cells (1). Next to T-LGLL, the 2016 World Health Organization classification of mature T- and Natural Killer (NK)-cell neoplasms, also lists the provisional entity of chronic lymphoproliferative disorders of NK cells (CLPD-NK) and aggressive NK-cell leukemia (ANKL) among monoclonal LGL proliferative diseases (2). T-LGLL, an expansion of CD3+ T-cell large granular lymphocytes (LGLs), is the most frequent variant representing ~85% of LGL proliferations and can further be subdivided into the common αβ-form and the rarer γδ-variant. Among the coreceptors CD8^+^ is usually more commonly expressed than CD4^+^. Additionally, mixed phenotype forms have been reported (3, 4). CLPD-NK accounts for approximately 10% and ANKL for approximately 5% of LGL proliferations. This case series excludes ANKL due to its clearly distinguished clinical and molecular features and different treatment approaches. We focus here on T-LGLL and CLPD-NK, both being referred to as LGLL.

The clinical presentation of LGLL is variable and typically includes cytopenias (particularly neutropenia and anemia), but often also symptoms of associated autoimmune disorders (mostly rheumatoid arthritis (RA), but also connective tissue diseases or vasculitis) (5). Furthermore, LGLL can be associated with secondary neoplasms, especially clonal B-cell expansions, but also solid cancers. The median patient age at diagnosis is 66 years, but approximately 15% of patients are younger than 50 years with an equal sex distribution (6, 7). Despite the course of LGLL being described as ‘indolent’, it is far from low-symptomatic and still associated with a shortened median overall survival (OS) of 9-10 years (8). Disease-related deaths are mainly due to severe infections. Such complications of the cytopenias and the autoimmune phenomena severely impair the quality of life of LGLL patients.

Diagnosis and management of LGLL is a challenge even for large academic centers. According to the WHO classification the diagnosis of LGLL requires a persistent (>6 months) increase in the number of peripheral blood (pB) LGL cells, usually 2-20 x 10^9^/L, without a clearly identified cause (2). Clonality is mandatory to be established and usually done by T-cell receptor (TCR) gene rearrangement studies (9, 10). In CLPD-NK monoclonality can indirectly be assessed by a restricted pattern of killer-cell immunoglobulin-like receptor (KIR) expression *via* flow-cytometric immunophenotyping, which is only done in few specialized laboratories. As a molecular hallmark, many LGLL harbor a genomic lesion of the signal transducer and activator of transcription 3 (*STAT3*). The gain-of-function *STAT3* mutations D661 and Y640 account for two-thirds of such variants (9). Additionally, variants of *STAT5B* have been recognized in a minority of T-LGLL cases. Both mutations cause constitutive activation of the JAK/STAT signaling pathway (9, 11). While former studies did not find a clear impact of these lesions on clinical outcome (9, 11), a recent retrospective single-center analysis of a large LGLL cohort found an independent association of *STAT3* mutations with shorter OS (4). More recently, missense mutations of the epigenetic regulator *TET2* were identified as another major genomic hallmark in CLPD-NK (12, 13).

Overall, the diagnostic pitfalls in LGLL range from recognition of the subtle T-cell or NK-cell proliferation, repeated establishment of their clonality, distinction of the LGLL clone from normal (T-) lymphocytes by a unique immunophenotype as well as detection and interpretation of molecular aberrancies in the context of a commonly normal karyotype. Additional diagnostic challenges are imposed by a coexisting RA or by laboratory findings of an autoimmune hemolytic anemia (AIHA) or of a pure red cell aplasia (PRCA) or of myelodysplasia. Problems in differential diagnosis also expand to the differentiation from related conditions such as Felty-syndrome or from other mature T-cell leukemias/lymphomas such as T-cell prolymphocytic leukemia (T-PLL) or hepato-splenic T-cell lymphoma (HSTL) (14).

With respect to its therapeutic management, LGLL is considered incurable by currently available options, including immunosuppressive agents and low-dose chemotherapy. Treatment-defining prospective trials are hardly available. For a summary of tested strategies see (15). Furthermore, there is a great deal of uncertainty regarding the optimal timing of treatment initiation.

Here we present four challenging and instructive cases of LGLL that presented to our centers with typical as well as rare features of this heterogeneous disease. This case catalogue serves to emphasize numerous diagnostic pitfalls, unique clinical scenarios, and various therapeutic modalities. Typical characteristics and special features are presented in **Table 1**.

**Table 1:** Characteristics and special features of presented patients.

	age	sex	LGLL phenotype	hepatosplenomegaly	cytopenia	autoimmune manifestations	hemoglobin (g/dl)	ANC (/µl)	platelets (/µl)	immunophenotype	percentage of LGLL cells (pB)	mutation	special features
**Patient 1**	62	m	CLPD-NK LGLL	yes	anemia thrombocytopenia	none	6.6	1692	62	CD2^+^CD16^+^CD7^+^CD3^-^CD4^-^CD6^-^CD8^-^CD56^-^CD57^-^	15.3%	*STAT3*	kidney donor
**Patient 2**	51	m	γδ-LGLL	yes	neutropenia	urticaria, hashimoto thyreoiditis	14.3	110	189	CD3^+^CD4^-^CD8^-^CD45RA^+^CD56^+^CD57^+^TCRγδ^+^	24%	*STAT3*	previous treatment with omalizumab
**Patient 3**	55	f	CLPD-NK LGL	no	anemia, neutropenia	None	7.7*	1230*	174*	CD2^+^CD3^-^CD4^-^CD8^+^CD7^+^CD57^dim^CD16^+^CD56^-^	82%	wildtype	alloHSCT 10 years prior to diagnosis
**Patient 4**	69	m	αβ / γδ T-LGLL	no	anemia, neutropenia	ulcerative colitis, pos. Coombs test	10.9*	1190*	231*	CD3^+^CD4^-^CD8^+^CD16^dim^CD57^dim^TCRα/β^+^ & CD3^+^CD4^-^CD8^-^CD16^+^TCRγδ^+^	74% & 16 % respectively	wildtype	PRCA

alloHSCT, allogeneic stem cell transplantation; ANC, absolute neutrophile count; CLPD-NK, chronic lymphoproliferative disease of natural killer cells; LGLL, large granular cell leukemia; n.d., not done; pB, peripheral blood; PRCA, pure red cell aplasia; PSA, Psoriasis arthritis; WBC, white blood cells. *values at last presentation, initial blood counts unknown.

## Cases

### Patient 1

A 62-year-old Caucasian male presented in 2019 with weight loss, transfusion-dependent anemia, and thrombocytopenia with bleeding-stigmata. Three years prior to diagnosis he developed a mild anemia without signs of hemolysis, but with detection of a population of atypical NK cells in pB. Two subsequent bone marrow (BM) examinations, however, did not show any signs of a hematological disorder. The relevant medical history included living kidney donation for his wife in 2012. Possible renal causes for the anemia were excluded.

In 2019, the repeated diagnostic work-up revealed immunophenotypic evidence of the aberrant NK-cell population (CD2^+^CD16^+^CD7^+^CD3^-^CD4^-^CD6^-^CD8^-^CD56^-^CD57^-^) both in pB and in BM. Next generation sequencing (NGS) detected a mutation in *STAT3* (variant allele frequency [VAF] 12%, c.1847 A>G p.E616G). By PCR a clonal TRG rearrangement was detected and cytogenetics showed a normal karyotype. Computed tomography (CT) scans revealed a hepatosplenomegaly, but no lymphadenopathy. All findings were consistent with the diagnosis of NK-LGL. Treatment with cyclophosphamide at 100 mg/d was initiated and after four weeks was reduced to 50 mg/d due to neutropenia.

After six months of therapy platelet counts had improved to 70,000/µl (from 35,000/µl). Hemoglobin (Hb) levels normalized after nine months of therapy. After 10 months both pB and BM showed no signs of infiltration by NK-LGL cells. Additionally the *STAT3* mutation could no longer be detected by NGS, implicating a molecular complete remission (CR). A [^18^F] Fluordesoxyglukose-positron emission tomography (PET-CT) confirmed a metabolic CR with normal spleen size. The patient is still in CR at one year after discontinuation of cyclophosphamide.

### Patient 2

A 51-year-old Caucasian male presented with splenomegaly and grade-4 neutropenia in December 2019. His medical history included chronic urticaria, which was previously treated with omalizumab, and a euthyroid Hashimoto thyroiditis.

Flow-cytometry of the BM aspirate showed an aberrant T-cell population with a CD3^+^CD4^-^CD8^-^CD45RA^+^CD56^+^CD57^+^TCRγδ^+^ phenotype. NGS revealed a mutation in *STAT3* (VAF 26%, p.G618R). Clonality of T-cells was demonstrated by consensus PCR consistent with the diagnosis γδ-T-LGLL.

The initial CT-staging revealed a splenomegaly without lymphadenopathy. Therapy with cyclophosphamide 100mg/d was initiated in January 2020, however, the dosage had to be reduced to 50 mg/d after 2 weeks. This therapy had eventually to be discontinued four months later due to worsening of neutropenia and repeated infections. Treatment was switched to cyclosporine with a targeted trough level of 150 ng/dl. Four weeks after initiation of cyclosporine, the absolute neutrophil count (ANC) started to increase and after two months on therapy a sustained improvement to a moderate neutropenia was detected.

Due to neuromuscular symptoms and exacerbated arterial hypertension, the targeted trough levels of cyclosporine were reduced to 100 ng/ml, which improved tolerance of the therapy. Six months after this the ANC had normalized. CT-based imaging further showed a normalization of spleen size and tapering of cyclosporine was started.

Flow cytometry of the BM aspirate at nine months after the start of cyclosporine showed a residual fraction of the aberrant T-cell population of 2.5% of the total lymphocyte count with the residual finding of mutated *STAT-3* at a VAF of 15%.

### Patient 3

A 55-year-old Caucasian female was diagnosed with a follicular lymphoma in 2002 that subsequently transformed into an aggressive B-cell lymphoma. After several lines of therapy, including fludarabine + rituximab (R), dexaBEAM (dexamethasone + BCNU+ etoposide + cytarabine + melphalan) followed by autologous stem cell transplantation, R+bendamustine, R-CHOP (cyclophosphamide + doxorubicin + vincristine + prednisolone) as well as radiotherapy, she received an allogeneic stem cell transplantation (alloHSCT) from a matched unrelated donor in December 2006 after conditioning with fludarabine and melphalan.

In November 2016 (10 years after alloHSCT), she developed neutropenia without signs of relapse or graft failure or infectious causes of myelosuppression. The underlying reasons could initially not be classified despite a thorough diagnostic work-up. Over the next two years she developed a transfusion dependent anemia and the diagnostic work-up was repeated. This time, flow cytometric analysis revealed an aberrant cell population (82% of total lymphocyte count) with the phenotype CD2^+^CD3^-^CD4^-^CD8^+^CD7^+^CD57^dim^CD16^+^CD56^-^ suggestive of a CLPD-NK. No mutations were found for *ATM*, *STAT3*, *STAT5b*, or *TP53* as per NGS studies. PCR analysis detected clonal *TRB* gene rearrangements. From these samples a complete donor chimerism was established, indicating the donor origin of the CLPD-NK. Due to renal insufficiency and prior exposition to cyclophosphamide, treatment with the JAK1/3 inhibitor Tofacitinib 11 mg/day, instead of methotrexate (MTX), was initiated. Under this therapy, the neutropenia improved from severe to mild within six weeks and hemoglobin levels stabilized above 10 mg/dl without further need of transfusions.

### Patient 4

A 69-year-old Caucasian male presented with anemia in September of 2005. Flow cytometry of pB showed a T-cell population with an immunophenotype (CD3^+^CD4^-^CD8^+^CD16^dim^CD57^dim^TCRα/β^+^) that was indicative of T-LGLL. Clonality of this aberrant T-cell population was proven by PCR. Cytogenetics showed a normal male karyotype and NGS revealed *STAT3* to be in wildtype configuration. His medical history included an IgG-lambda-monoclonal gammopathy of undetermined significance, ulcerative colitis, and coronary artery disease. Therapy with MTX (initially 10 mg, increased to 15 mg in March 2006) was initiated in 2006 due to declining hemoglobin levels. In 2007 a complete remission (CR) was documented, but the patient relapsed four months after discontinuation of MTX. Further therapy with four courses of fludarabine (25 mg/m2 day 1-3 every 28 days) was initiated and resulted in a second CR, which lasted for seven years until January 2014. At that time, therapy with fludarabine was repeated and the patient again achieved a clinical response that lasted until May 2019.

In June 2016, flow cytometry of pB revealed a second aberrant T-cell population, accounting for 17% of lymphocytes and presenting with the following phenotype: CD3^+^CD4^-^CD8^-^CD16^+^TCRγδ^+^. In 2019 the patient experienced another relapse with a lymphocytosis of 5900/µl and by subsequently developing symptomatic anemia. Another cycle of fludarabine was initiated in May 2020, but without improvement in hemoglobin levels. As Coombs tests were positive, suggesting an autoimmune hemolytic etiology of the anemia, a therapeutic attempt with prednisolone (maximal dose 75 mg and subsequent tapering), followed by initiation of tofacitinib for three months, was made, but neither resulted in improvements. The trephine BM biopsy showed an isolated absence of erythropoiesis without detection of infiltration of T-LGLL cells, fulfilling the criteria of a PRCA. Treatment with cyclosporine and prednisolone was initiated in April 2021, resulting in an ongoing clinical response with stable hemoglobin levels and without further need for transfusions.

## Discussion

Here we present heterogeneous presentations of T-LGLL and CLPD-NK that were seen in two academic institutions, with a focus on their diagnostic and therapeutic challenges. Our patients presented with unspecific symptoms, i.e. splenomegaly, autoimmune-mediated findings, or symptoms of cytopenias with a coincidental detection of LGL cells in flow cytometry.

In LGLL, often low-level lymphocyte infiltrations are misinterpreted as reactive, which frequently delays the definitive diagnosis. A thorough algorithm in the context of a fitting set of clinical presentations should include cytomorphology/histology, flow cytometry, a molecular clonality analysis, and gene-sequencing studies (11, 14, 16, 17).

The most common phenotype of T-LGLL is CD3^+^CD4^neg^CD5^+/low^CD8^+^CD16^+^CD57^dim^. However, neither the described immunophenotype nor the morphological features of LGLs are entirely specific (14). Consequently, an accurate distinction from other mature T-cell disorders, e.g. early-phase (low proliferative) T-PLL, is highly relevant, prognostically and therapeutically, but can sometimes be difficult and requires the incorporation of diagnostic multi-parameter approaches. A prolymphocytic morphology is only found in ~60% of T-PLL and the post-thymic pan-T (CD2^+^CD3^+^CD5^+^CD7^+^) immunophenotype of T-PLL includes in a small fraction of cases also the T-LGLL-like CD4^-^CD8^+^ pattern (14, 18). However, detection of a locus rearrangement involving a *TCL1* gene (either TCL1A at chromosome 14 [mostly as an inv (14)] or *MTCP1* at chromosome X) or proof of TCL1 protein expression in T-cells are established as unique major diagnostic criteria for T-PLL (14, 19–21)

CLPD-NK typically shows a CD3^-^CD56^+^CD57^+/-^ immunoprofile. Cases of CD56^-^ CLPD-NK, as displayed in patients 1 and 3, have been described as well (22). CLPD-NK is associated with rather indolent courses and less often symptomatic than T-LGLL. Cytopenias and infections are characteristic as well as a higher incidence of second neoplastic diseases, however, the latter is observed across all subsets of LGLL.

There is a known association of LGLL with autoimmune conditions (23). Approximately one third of patients with LGLL suffer from rheumatoid arthritis (24) and less frequently from other autoimmune diseases like systemic lupus erythematosus, Sjögren syndrome, or autoimmune thyroid disorders (5). This association seems to be far less present in CLPD-NK (25). Consistent with the literature, patient 2 of our series had a history of chronic skin allergies and thyreoiditis while patient 4 suffered from ulcerative colitis.

Interestingly, one of our two CLPD-NK cases (patient 3) arose after an alloHSCT and the tumor cells were of donor origin. Self-limiting proliferations of LGLs after alloHSCT without clinical relevance have been described previously (26, 27), but aggressive forms of LGLL from donor cells were reported only in single case-reports (28–30) and in a series of four patients (31).

Of note, in both cases of CLPD-NK presented here, clonal TCR gene rearrangements were detected by PCR. This may seem contradictory, however, an analysis of KIR-restricted CLPD-NKs revealed TCR rearrangements in 50% of patients at first diagnosis (32).

We further presented a case of γδ T-LGLL (patient 2) and a case (patient 4) with a mixed phenotype of αβ/γδ LGLL. Interestingly, patient 4 developed the γδ clone during the course of the disease. In a recently published series of LGLL, the γδ variant accounted for approximately 15% of cases (4). Cases of αβ/γδ mixed phenotype are extremely rare and have so far been reported only as isolated cases or in very small series (3, 4). The diagnosis of γδ T-LGLL can be challenging; especially the differentiation from HSTL, which is often of γδ type and typically shows a low-level leukemic presentation. Particularly difficult are LGLL cases with absent or very low counts of clonal LGLL cells in pB and/or with a CD4-/CD8- phenotype (33). Detection of cytogenetic aberrations (isochromosome 7q or trisomy 8), described in >50% of HSTL (34), but atypical for T-LGLL, can be of assistance. HSTL affects predominantly younger men typically in the context of (medical) immunosuppression, often for a pre-existing autoimmune disease, yet the HSTL itself has not been associated with autoimmune phenomena. Nevertheless, the invariably aggressive course of HSTL sets it well apart from LGLL.

Another challenge is to define the cause(s) of cytopenias in LGLL, especially when these can also be caused by the therapeutic strategies. Patient 4 presented with anemia and underwent treatment with MTX and fludarabine. After becoming refractory to this treatment, further diagnostic workup was necessary to discriminate between therapy-related BM toxicity, AIHA, and PRCA, and eventually the diagnosis of PRCA was made. The association of LGLL with both AIHA (1, 17, 35) and PRCA (36) has been described and the accurate discrimination of those entities is necessary for the choice of an effective therapy (37, 38).

Although most LGLL patients do not require treatment at presentation, in 2/3 of cases therapy needs to be initiated at a later stage, especially due to severe neutropenias and subsequent infections, transfusion-dependent anemias, or thrombocytopenias (24). The current treatment options in LGLL are extremely limited. Standard approaches are based on supportive measures (e.g. transfusions, hematopoietic growth factors, antibiotics) and immunosuppressive therapies like MTX, cyclophosphamide, or cyclosporine (16) with limited evidence. The optimal sequence of MTX and cyclophosphamide is yet to be determined (ongoing trial NCT01976182).

Cyclophosphamide seems to show better efficacy in the control of symptoms and cytopenias as compared to MTX, but due to associated late toxicities it should not be administered for more than 12 months (39). Both agents need a minimum of 6-12 weeks before definite response assessments (17) and they both have treatment-associated cytopenias as side effects. In our case series, cyclophosphamide was administered in two patients and proved to be an active treatment option even for a living kidney donor. Reports on responses to other substances, like rituximab (40, 41) or the JAK1/3 inhibitors tofacitinib (42) and the JAK 2 inhibitor ruxolitinib (39) are sporadic and limited to small series. Generally, the treatment responses in LGLL are usually dissatisfactory being frequently incomplete and/or short-lived.

Another relevant aspect is the disease-inherent and treatment-related immunosuppression, which is of particular focus in current contexts of the COVID-19 pandemic. Although here not represented with a particular case, it has been repeatedly shown that patients with hematologic malignancies have a higher risk of severe or fatal COVID-19 infections (43–45). A single center retrospective analysis of 835 patients hospitalized with COVID-19, recently showed a significantly increased mortality of patients previously receiving immunosuppressive therapies (46). However, due to the rarity of LGLL, the exact morbidity and mortality risks related to COVID-19 infections in LGLL patients are unknown. Moreover, we do not know how disease and immunosuppressive therapies influence the effectivity of anti-COVID-19 vaccinations in LGLL patients. In other lymphatic malignancies, such as chronic lymphocytic leukemia (CLL), responses to vaccination are influenced by disease activity, current treatments, and previous therapies, especially regarding anti-CD20-antibodies (47, 48). A pragmatic strategy might be to adopt vaccination strategies from other hematologic malignancies like CLL, but not to neglect complex aspects of both humoral and cellular immunity specific for LGLL (49).

In summary, LGLL is a heterogenous group of diseases ranging from asymptomatic presentations over cases with severe impairments of quality of life, but long survival, to cases with significantly shortened life expectancy. In this series of selected cases with unique features, we illustrate pitfalls in the diagnosis, management, and treatment of LGLL of T-cell and NK-cell nature. In agreement with the literature, the uniqueness of the individual presentations and courses seems to override potential associations of clinical features, treatment responses, or outcomes with phenotype (αβ vs. γδ or T vs. NK) or genotype.

## Data Availability Statement

The raw data supporting the conclusions of this article will be made available by the authors, without undue reservation.

## Ethics Statement

Written informed consent was obtained from the individual(s) for the publication of any potentially identifiable images or data included in this article.

## Author Contributions

MH, NP, and VV contributed to conception and design of the study. AL, AS, DB, LW, NP, and VV acquired patient data and organized the database. NP and VV wrote the first draft of the manuscript. AL, AS, DB, and TB wrote sections of the manuscript. AD, AM, DJ, EB, MO, and TB provided diagnostic support. G-NF, MJ, SS, and UP provided administrative support. All authors contributed to the manuscript revision, read and approved the submitted manuscript.

## Funding

The authors acknowledge support from the German Research Foundation (DFG) and Universität Leipzig within the program of Open Access Publishing” or “Dieser Artikel wurde durch den Publikationsfonds der Universität Leipzig und das Programm Open Access Publizieren der DFG gefördert”.

## Conflict of Interest

The authors declare that the research was conducted in the absence of any commercial or financial relationships that could be construed as a potential conflict of interest.

## Publisher’s Note

All claims expressed in this article are solely those of the authors and do not necessarily represent those of their affiliated organizations, or those of the publisher, the editors and the reviewers. Any product that may be evaluated in this article, or claim that may be made by its manufacturer, is not guaranteed or endorsed by the publisher.
